# Influence of Concussion History and Genetics on Event-Related Potentials in Athletes: Potential Use in Concussion Management

**DOI:** 10.3390/sports6010005

**Published:** 2018-01-19

**Authors:** Taylor Guth, Caroline J. Ketcham, Eric E. Hall

**Affiliations:** 1 College of Science, University of Notre Dame, Notre Dame, IN 46556, USA; tguth@nd.edu; 2 Department of Exercise Science, Elon University, 2525 Campus Box, Elon, NC 27244, USA; cketcham@elon.edu

**Keywords:** concussion, neurocognitive performance, genetics, cognitive function, mild traumatic brain injury, concussion management

## Abstract

Sports-related concussions are an increasing public health issue with much concern about the possible long-term decrements in cognitive function and quality of life that may occur in athletes. The measurement of cognitive function is a common component of concussion management protocols due to cognitive impairments that occur after sustaining a concussion; however, the tools that are often used may not be sensitive enough to expose long term problems with cognitive function. The current paper is a brief review, which suggests that measuring cognitive processing through the use of event related potentials (ERPs) may provide a more sensitive assessment of cognitive function, as shown through recent research showing concussion history to influence ERPs components. The potential influence of genetics on cognitive function and ERPs components will also be discussed in relation to future concussion management.

## 1. Introduction

The Centers for Disease Control and Prevention estimates that as many as 3.8 million sport and recreation-related concussions occur each year in the United States [1]. Sports-related concussion (SRC) is caused by a direct blow to the head, face, neck, or other area of the body that sends an impulsive force to the head [2]. SRC is mainly classified by rapid onset of temporary, mild impairment of neurological function. The injury is predominantly functional, with no apparent structural damage detectable with neuroimaging techniques. Clinical symptoms may develop up to hours after the injury and are typically transient. In some cases, loss of consciousness may occur [2].

Although concussion has been considered a transient injury, a number of recent studies link multiple concussions with long-term neurodegenerative issues [3,4,5]. One of these effects is Chronic Traumatic Encephalopathy (CTE), which refers to neurocognitive decline and neuropathological findings of the accumulation of atypical hyperphosphorylated-tau neuronal deposits in brain tissue [6]. CTE has been detected in contact-sport athletes, such as football, ice hockey, and wrestling [7,8]. A 2017 study found neuropathologically diagnosed CTE in the brains of 110 out of 111 deceased National Football League players [9].The prevalence and risk factors of CTE are yet to be understood, and more research must be done to determine the relationship between CTE and concussion history [6].

The prevalence and potential lifelong effects of concussion demand that more attention must be directed towards continually improving concussion management in an effort to minimize short and long-term injury. Proper concussion management allows for safe and effective return-to-play decisions to be made. This reduces the chances of exacerbating a current concussion, which, as shown by CTE research, may lead to serious long-term brain dysfunction. Current clinical concussion management protocol for athletic trainers includes neurocognitive evaluation [10]. The Immediate Post-Concussion Assessment and Cognitive Test (ImPACT) is a widely-used computerized concussion diagnostic tool. However, studies have suggested that the ImPACT fails to show, underlying neurocognitive deficits that may exist [11]. The detection of more subtle neurocognitive deficits may be better suited to highly sensitive electrophysiological measures such as the electroencephalogram (EEG). EEG technology is used to record brain activity through electrodes placed on specific locations of the scalp. When an individual performs a cognitive task, the EEG records the neural activity as a series of waveforms. This scalp-recorded neural activity is referred to as event-related potentials (ERPs). The ERPs are generated in a neuroanatomical module when a specific computational operation is performed and correspond to voltage deflections of relatively independent latent components [12].

Some studies suggest that cognitive ERPs, which can be used to assess a variety of cognitive processes ranging from visual attention (P1, N1) to spatial analysis (N2) and visual perception (P3), can be influenced by genetics [13,14,15,16]. Reinvang et al. conducted a study that concluded that cognitive ERPs are linked to the variation in the alleles for the Apolipoprotein E gene in mildly cognitively impaired patients [15]. This gene has been studied heavily in relation to CTE and Alzheimer’s Disease (AD), and more recently in relation to concussions and performance on neurocognitive tests such as the ImPACT [17,18]. Relatively few studies exist that examine the connection between ERPs, genetics, and concussions. Evaluating the relationship between these three factors may provide greater insight into neurocognitive performance in athletes both prior to and following a concussion. A better understanding of this interaction may enhance clinical management of concussed athletes by providing more details about the current technology used to evaluate concussions. Further, it may lead to more knowledge about how a patient’s genotype may affect performance on post-concussion evaluations. The purpose of this brief review will be to examine the evidence that supports the use of ERPs in detecting short and long-term decrements in cognitive processing that occur and to examine the possible influence of genetics on these markers.

## 2. Review of Event Related Potentials

The major ERP components include N1, Mismatch Negativity (MMN), the N2 family, the P3 family, and Error Related Negativity (ERN) [12]. The N1 component is generated in the auditory cortex on the dorsal side of the temporal lobes and is classified by a frontocentral component that peaks near 75 ms. N1 has a vertex-maximal potential that peaks near 100 ms and a laterally distributed component that peaks around 150 ms, and comes from the superior temporal gyrus and is attention-sensitive.

The MMN component appears when an individual has been attending to a repetition of identical stimuli and then is presented with an out-of-the ordinary stimulus. The MMN peak component is indicative of a process that compares the most recent stimuli to the sensory memory trace of previous stimuli. MMN is observed as a negative wave that is maximum size at central midline scalp sites and peaks between 160–220 ms.

N2 is the second negative wave peak of an ERP and occurs between 200 ms and 350 ms following the presentation of a stimulus [19]. An N2 deflection occurs with the onset of a repetitive, non-target stimulus. When deviant stimuli are presented, N2 amplitude increases, and this basic N2 deflection likely contains other components; Task-irrelevant deviant stimuli are thought to elicit the N2a peak (appearing first in the N2 latency range), while task-relevant deviant stimuli elicit the N2b peak.

The P3 component corresponds to the devotion of attention to significant environmental events, and records the constant refreshing of working memory [20,21,22,23]. P3 amplitude is greater when a task requires more effort, suggesting that P3 amplitude is a measure of resource allocation. Further, P3 amplitude is dependent on stimulus probability—the lower the probability of the stimulus, the greater the amplitude and the eliciting stimulus task relevance. P3 latency is thought to reflect the time necessary to evaluate the stimulus [24,25].

**Figure 1:**
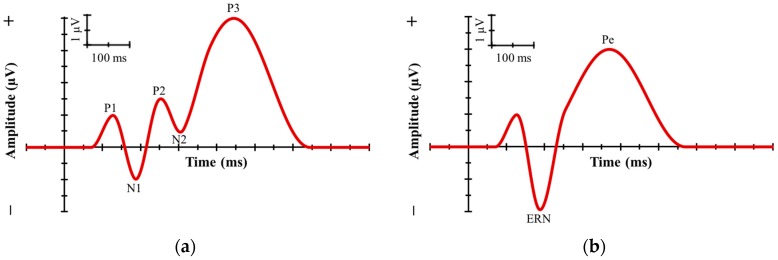
Examples of ERP components. Figure 1a shows common stimulus-locked components of the ERP. Figure 1b demonstrates stimulus-locked components of the ERP. ERN = Error Related Negativity; Pe = Error Related Positivity (Figure reprinted with permission from Nova Science Publishers, Inc. Reprinted from: Concussion in Athletics: Assessment, Management and Emerging Issues, by Alderman, Bixby & Olson, Chapter 9: Using Event-Related Potentials to Assess Neurocognitive Impairments Following Sports-Related Concussions, **2017, ** pp. 127–142.) [28].

Two ERP components that are stimulus-locked are the ERN and the Error Related Positivity (Pe). The ERN is a peak component characterized by a negative-going deflection at the frontal and central electrode sides following the time of response. It is generally thought that the ERN corresponds to the system that monitors responses or is sensitive to conflicts between intended and actual responses [19,26]. The Pe, a less studied ERP component, appears following the ERN on error trials and is thought to be used in error monitoring [26,27]. See Figure 1 below for a visual of these representative ERP components.

## 3. Concussion History and Event-Related Potentials

For more comprehensive reviews of the literature regarding ERPs and their use in concussion, please see the reviews by Alderman et al. and Broglio et al. [28,29]. A study by Broglio et al. compared the ERPs of a group of young adults with a concussion history to a group without a concussion history (N = 90, 19.7 ± 1.3 years: 44 without concussion and 46 with previous concussion)—a novelty oddball task [30]. The results showed decreased N2 and P3b latencies in the group with a concussion history relative to the control group. This same sample was used in another study that used the Flanker test to measure the ERN [31]. The concussion group showed suppressed ERN responses to errors committed. More significantly, as the number of concussions increased, the ERN response decreased. Because those individuals with a concussion history were less likely to acknowledge when they had committed an error, these results suggest that persistent electrophysiological effects of concussion may exist. The findings of impaired P3 and N2 components has also been found in other studies as well [32,33,34].

Gaetz et al. studied the effects of multiple concussions in groups of hockey players using N2/P3 and CNV (Contingent Negative Variation) paradigms [35]. The results demonstrate that players with three or more concussions differed significantly on several cognitive post-concussion symptoms. These players also differed significantly for the latency of the P3 response when compared to those with no concussion history. Suppressed P3 amplitude in athletes with multiple concussions was also documented in a study by De Beaumont et al. [36]. Similarly, those who have suffered three or more concussions have been found to have a greater sustained posterior contralateral negativity and impaired working memory [37].

Ledwidge and Molfese found larger N2/P3 amplitudes in athletes with multiple concussions and longer P3b latencies [38]. A recent study by Moore et al. analyzed cognitive function in collegiate male athletes who have sustained a concussion participating in contact sports [39]. Collegiate male athletes in non-contact sports who have not sustained a concussion composed the control group. Using neuropsychological tests and a three-stimulus oddball task, they found that concussed or sub-concussed athletes displayed delayed recall deficits and concluded that concussive and sub-concussive injuries are consistent with cognitive deficits. They also found that P3a, P3b, and N1 amplitudes were decreased in sub-concussed and concussed athletes compared to controls. The results of these studies support the hypothesis that individuals with a past concussion recruit compensatory neural networks to meet critical needs of executive functioning. In addition to collegiate athletes, similar findings have been found in older populations [40] and younger populations [41,42] that have previously suffered a concussion.

These waveforms have also been connected to post-concussion symptoms. In a study done by Lavoie et al., P3b amplitude and symptomatology were inversely related, as symptomatic athletes had smaller P3b amplitudes relative to asymptomatic athletes [43]. In this study, symptom severity was determined using a concussion symptom scale that consisted of 19 of the most common post-concussion symptoms (e.g., headache, dizziness, and trouble falling asleep). Lavoie et al. concluded that the degree to which symptoms present could indicate the existence of post-concussion functional deficits.

The studies detailed above demonstrate the ability of event-related potentials, specifically that of the P3b and N2 waveforms, to detect cognitive dysfunction in those with a history of concussion. Because P3b amplitude is associated with the allocation of attentional resources to a stimulus, it is possible that the amplitude is a function of the amount of resources being employed at a specific point in time following stimulus presentation. It then follows that a decrease in amplitude post-concussion indicates that the ability to recruit neural networks needed to attend to a stimulus has been impacted. Overall, these findings suggest that concussion history may affect the amplitude or latency of ERP components, and thus EEG may prove a reliable technology for detecting and measuring underlying cognitive deficits. Impairments in cognition are often seen acutely following a concussion, and long-term deficits in cognitive function as a result of concussion are a major concern. The use of commonly used neurocognitive assessments, such as ImPACT, may not be sensitive enough to detect these changes [11]. Therefore, the examination of various ERP components maybe a useful technique for future examination of these impairments. However, the utility of ERPs while recovering from a concussion and going through stepwise progressive exercise to return-to-play has not been investigated.

## 4. Neurocognitive Tests and Concussion Management

One of the most widely used computerized neurocognitive tests in concussion diagnosis and management is the ImPACT [44]. ImPACT generates composite scores for verbal memory, visual memory, visual motor processing speed, and reaction time, according to six neurocognitive modules that target various aspects of cognitive functioning such as memory, attention, processing speed, and reaction time [45]. This test is generally accepted as a useful diagnostic and management measure, as the various paradigms make it difficult for patients to experience the practice effect [46].

In 2001, the first International Conference on Concussion in Sport held in Austria deemed neurocognitive testing the paramount concussion diagnostic tool and placed much promise in computerized neurocognitive test batteries regarding detection of concussions [47]. However, the conference committee speculated about the sensitivity and reliability of such tests, calling for more research to be done. A study by Teel et al. demonstrated that concussed participants were able to pass clinical concussion testing tools, such as the ImPACT, within 8 (5 ± 1) days after injury [11]. Due to the precision of EEG measures, a reduction in EEG power in concussed individuals compared to those in the non-concussed group was detected. This suggests that concussed individuals recruit additional brain networks to perform a task, making cognitive processes more effortful. While the ImPACT may be a useful neurocognitive evaluation that currently exists for its ability to measure a variety of cognitive functions and its non-repetitiveness, it is not an infallible method of diagnosing and managing a concussion, as the sensitivity of this test appears to decrease around one week post-injury. To best assess and diagnose an athlete following a head injury, it may be effective to employ electrophysiological measures, such as ERPs, in addition to neurocognitive testing.

## 5. Influence of Genetics on Neurocognitive Performance and ERPs

The 2001 International Symposium on Concussion in Sport first expressed interest in the manifestations of neurocognitive deficits in relation to genetic phenotype [47]. This concern is yet to be resolved, as the 2016 international conference on concussion affirmed that genetic testing for traumatic brain injury (TBI) currently has limited application to SRC, yet interest still exists regarding the role of genetics in predicting the risk of initial injury, duration of recovery, long-term neurological function following SRC, and concussion history [2]. It has been suggested that cognitive ERPs have genetically-influenced characteristics [48]. In particular, twin studies have demonstrated the potential effect of genetic factors on EEG. In a study of genetic variability in resting EEG in monozygotic and dizygotic twins, no consistent differences were seen in the EEG output of monozygotic twins, unlike that of dizygotic twins. The EEG of monozygotic twins were similar with respect to persistence, amplitude, frequency, and alpha index. The study concluded that heredity alone determines EEG variability, and that EEG relies on a multifactorial genetic system [49,50]. The genes of particular interest for their potential effect on event-related potentials are Apolipoprotein E, Catechol-O-methyltransferase, and Dopamine Receptor D2, as they have been previously suggested to influence concussion susceptibility and cognitive function [51].

### 5.1. Apolipoprotein E Gene

One genetic factor of particular interest is the allelic variation of the Apolipoprotein E (ApoE) gene. The ApoE gene on chromosome 19 is essential in plasma lipid metabolism. The ApoE gene has three major isoforms: e2–e4 [52]. The vast majority of scientific focus surrounds the e4 (epsilon4) allele, as its presence may affect cognitive processing [17].

Specifically, the e4 allele has been associated with an increased risk for developing Alzheimer’s Disease (AD), and, when coupled with a history of head injury, the risk of Alzheimer’s Disease may increase ten-fold [53]. Scientific inquiry is also interested in potential connections between the ApoE gene and other neurological diseases, including CTE. In a study of twelve neuropathologically confirmed cases of CTE, five (42%) cases were ε4 carriers, two (17%) of which were homozygous for the ε4 allele [4]. Other studies have used genetic testing along with neuropathological examinations of individuals with history of head trauma to conclude that approximately 57% of individuals with neuropathologically-confirmed CTE possessed at least one APOE ε4 allele [54]. In contrast with the estimated 28% of the population with at least one APOE ε4 allele present, the frequency of this allele in those with CTE appears higher than expected [55] However, it must be noted that e4 individuals in these studies that link the APOE gene to CTE comprise a selective sample and may be misrepresentative of the general population. More research must be done before this gene can be a proven marker for CTE susceptibility.

The e4 allele of the ApoE gene is also associated with decreased performance on tests of global cognitive function and episodic memory [56,57,58]. Nilsson et al. [57] found that carriers of two e4 alleles failed more profoundly in the acquisition and recollection of episodic information than did carriers of one e4 allele, who failed more than non-carriers of the e4 allele.

Crawford et al. (2002) linked the e4 allele to deficient memory performance and rehab outcome following a TBI. Athletes who have suffered an mTBI (mild traumatic brain injury) and who carry the e4 allele are more likely to experience post-concussion headache and report more severe post-concussion headache symptoms than concussed athletes who lack the e4 allele [59]. Subcommittees of the American Academy of Neurology have suggested that the e4 allele of the ApoE gene is a risk factor for chronic neurobehavioral impairment following a concussion and should be examined further [60].

In addition to cognitive function, the e4 allele has been associated with lower N2 amplitude and may be indicative of a gene-dose relationship, with e4 homozygotes exhibiting lower amplitudes than e4 heterozygotes [15]. A study done by Espeseth et al. showed that e4 homozygotes had longer N2 latencies compared to carriers of only one e4 allele (e3/e4) and e3 homozygotes [61]. ApoE genotype may be linked to cognitive impairment in individuals through the use of ERP. N2 and P3 latencies increased in cognitively impaired patients with the presence of ApoE e4 allele [62]. Green and Levey found that individuals with multiple risk factors for Alzheimer’s disease, such as family history and the e4 allele, produce significantly longer auditory N2 and P3 latencies than those with family history of AD but no ε4 allele [63]. Further, the P3 amplitude of amnestic mild cognitive impairment (aMCI) patients has demonstrated exacerbated visuospatial working memory deficits in e4 carriers in comparison to e4 non-carriers [64].

A specific single-nucleotide polymorphism (SNP) in the ApoE gene (rs405509) is commonly known as the APOE Promoter. This SNP is characterized by a T allele in lieu of a G allele, and is linked to increases concussion susceptibility ([65,66]). Individuals who are homozygous for the T allele were three times more likely to have a history of concussion as opposed to those homozygous for the G allele of the ApoE promoter. Furthermore, the possession of a T allele with the e2 or e4 isoform of the ApoE gene may exacerbate concussion susceptibility in collegiate student-athletes [65].

### 5.2. Catechol-O-Methyltransferase Gene

The catechol-O-methyltransferase (*COMT*) gene is responsible for the breakdown of dopamine and serotonin in the brain. The presence of the *COMT* Met allele may increase performance on tasks of executive functioning [67,68,69,70] and working memory [70,71,72]. A study by Yue et al. using a small population sample demonstrated that individuals with the Val/Met genotype exhibit poorer performance, lower P300 amplitude, and higher P300 latency than those bearing the Val/Val genotype in a continuous performance task with visual stimuli [73]. Other investigations have shown that individuals possessing the Val allele of the *COMT* gene have extended P300 latencies [74,75]. However, other studies provide contradictory results and have not found COMT to influence ERP components [13,16]. Despite these varying results related to ERPs, the COMT gene has been found to be related to neurocognitive performance on a baseline concussion assessment [17] and may be promising for studying the impact of genetics on ERPs.

### 5.3. Dopamine Receptor D2 Gene

It has been suggested that the dopamine receptor D2 (DRD2) be examined in relation to concussions, because it has been hypothesized to effect executive function and possibly be related to Alzheimer’s disease [51]. A study done by Lin using an auditory oddball paradigm found no association between DRD2 and P300 latency [14]. Further examination of the influence of DRD2 as a candidate gene exploring relationships between executive function and concussion history, although much further work would be needed.

More research must be done on the effects of ApoE, COMT, and DRD2 on ERP before EEG technology can be used in conjunction with genetics as a concussion evaluation and management tool. Additionally, research is needed to demonstrate that there is a link between concussion history, genetics and ERP components, and until this link is made, any recommendations should be made with caution. However, if these relationships can be confirmed, these genes could serve as useful biomarkers in concussion management for their potential relation to cognitive function.

## 6. Conclusions

In order to confirm the relationships hypothesized in this paper, future studies could focus on incorporating possibly more sensitive measures of cognitive function such as those provided by ERPs and genetics into the diagnosis and management of sports-related concussions, but also may be important in determining long-term implications of concussions in athletes. Studies could be conducted that examine ERP components during recovery and while returning-to-play. Additionally, a more thorough understanding of the influence of type of task and the ERP components elicited need to be undertaken. McDevitt & Krynetskiy have suggested genotyping of athletes as a potential method for clinicians to improve concussion management and return-to-play decisions [76]. Specifically, studies could be done regarding the COMT and DRD2 as potential candidate genes in relation to concussion, as previous studies have linked these genes to significant anomalies in cognitive function and ERP components [13,17,73,74,75]. Moreover, the alleles of the ApoE gene, specifically e4, could be further researched for an effect on post-concussion cognitive dysfunction due it their potential connection to Alzheimer’s disease and CTE. Studies suggest that the presence of the e4 allele may affect baseline neurocognitive performance as measured by the ImPACT [17]. However, few studies, if any, exist that have examined the presence of the e4 allele on neurocognitive performance using tests like ImPACT and ERP following a concussion. This method would offer further insight into neurocognitive functioning on a thorough and specific basis. If specific alleles can be associated with performance on ImPACT and ERP, concussion diagnosis and management could be better predicted and possibly tailored to individuals of certain genotypes.
